# Epitope Analysis of Cerebrospinal Fluid IgG in Japanese Multiple Sclerosis Patients Using Phage Display Method

**DOI:** 10.1155/2011/353417

**Published:** 2011-11-10

**Authors:** Juichi Fujimori, Ichiro Nakashima, Kazuo Fujihara, Tatsuro Misu, Shigeru Sato, Yasuto Itoyama

**Affiliations:** ^1^Department of Neurology, Tohoku University School of Medicine, 1-1 Seiryo-machi, Aoba-ku, Sendai 980-8574, Japan; ^2^Department of Neurology, Kohnan Hospital, Sendai 982-8523, Japan

## Abstract

To investigate the antigen recognized by cerebrospinal fluid (CSF) high affinity IgG in patients with multiple sclerosis (MS), the phage display method was applied to the CSF from 15 MS and 10 control patients. Peptide sequences recognized by MS and control CSF IgG were individual specific, and no common motif was found. Peptide sequences frequently showed homology to various kinds of amino acid sequences of ubiquitous viruses such as epstein barr virus (EBV) and herpes simplex virus (HSV), although the frequency was not specific to MS patients. MS CSF IgG may recognize various types of ubiquitous viral antigen and may be increased by a bystander response.

## 1. Introduction

In multiple sclerosis (MS), the pathogenetic events that result in immune cell infiltration, multifocal demyelination, and axonal loss are still under debate. Recognition has emerged that both mutually interacting cellular and humoral immune components may contribute to immune-mediated demyelination in human disorders [1]. 

Normal cerebrospinal fluid (CSF) has an IgG content that is less than 13% of the total protein. In MS, there is an unexplained elevation of IgG in the CSF from 15% to 30%, visualized as oligoclonal bands (OB) after electrophoresis [2].

Although the humoral immune response is implicated in MS, its role in the pathogenesis has not been determined precisely. Most researchers seem to consider MS CSF IgG or oligoclonal IgG band to be a nonspecific bystander response. On the other hand, several laboratories studied the antigen-binding regions of antibodies found in MS brain demyelinative plaques and cerebrospinal fluid and revealed (a) limited germline expression, results not expected for a random bystander response; (b) features consistent with a specific antigen-targeted process; (c) the clonal expansion of populations of B lymphocytes in MS [2]. Furthermore, the target antigen of MS CSF IgG has not been determined.

 We have successfully confirmed that CSF IgG from HTLV-I-associated myelopathy/tropical spastic paraparesis (HAM/TSP) patients selected motifs that were highly homologous to the causative microorganism “HTLV-I” from a 12-mer random peptide library (RPL) using the phage display method [3]. We tried to find the target antigen of CSF IgG in MS patients using the same method as we did in the cases of HAM/TSP.

## 2. Materials and Methods

### 2.1. Patients and Controls

CSF samples from 15 MS patients (13 women and 2 men, mean age 37 (range, 21–60) years,) were analyzed. The mean onset age of the patients was 29 years, and the mean duration of the disease was 8 years. The mean IgG index was 1.07. All MS patients fulfilled McDonald's criteria and the samples were obtained during acute relapses of the disease. By testing with isoelectric focusing and IgG immunofixation method, 14 patients were found to be OB positive and one patient (MS7) was OB negative. In a single patient (MS6), CSF samples collected at three different points of time were studied. 

The control group was comprised of 5 patients with psychosomatoform disorder and 5 patients with headache. CSF samples from 10 control patients (5 men and 5 women), mean age 28.7 years (range, 14–48), were analyzed.

### 2.2. Phage Display Method

One hundred microliters of Dynabeads Protein A (Dynal Biotech ASA, Oslo, Norway) were incubated with 300 *μ*L of CSF overnight at 4°C. The IgG-bead complexes were incubated with 10 *μ*L of a 12-mer random peptide library containing approximately 4 × 10^10^ particles (Ph.D-12, Phage Display Peptide Library Kit, New England Biolabs, Beverly, MA) for 60 minutes at room temperature. After washing twice with phosphate-buffered saline containing 0.1% Tween-20, bound phages were eluted and amplified by infecting Escherichia coli (ER2738 strain) and incubated for 4.5 h at 37°C. Bacterial cells were removed by centrifugation, and the amplified phages were purified by polyethylene glycol (PEG) precipitation. The phage solution was titrated to determine the concentration. Newly made IgG-bead complexes were mixed for 60 minutes with the 2 × 10^11^ phage particles derived from the first round phage selection. After washing six times, bound phages were eluted. Thirty-three clones from the second round of phage selection were sequenced for DNA to determine the displayed 12-mer amino acids. All these procedures were based on the manufacturer's protocol. The selected phage clones were numbered in each patient. For example, p7_33 denoted the 33rd clone in patient MS7.

### 2.3. Homology Search for Selected Amino Acid Sequence Motif

We searched for a common peptide motif, a specific sequence pattern that occurs repeatedly in a group of peptide sequences, using Multiple Expectation Maximum for Motif Elicitation (MEME) version 3.0 (http://meme.sdsc.edu/meme/cgi-bin/meme.cgi/)  [4].  BLAST (Search for short nearly exact matches, National Center for Biotechnology Information) was searched for proteins containing homologous sequences. 

## 3. Results

### 3.1. Phage Display

CSF from 13 out of 15 MS patients and CSF from 8 out of 10 control patients selected one or more common peptide motifs, but the peptide motifs were basically different from one patient to another (Tables 1 and 2). We tried to detect common peptide motifs in a total of 495 motifs derived from 15 MS patients, but we could not detect any MS-specific common peptide motifs.

### 3.2. Homology Search

In the BLAST search, we could not detect microbial agents or human molecules that significantly correlated with MS patients. However, we focused on non-human sequences with the intention of obtaining insight into etiological microbial agents of MS. The detected peptide motifs were frequently homologous to the components of herpes simplex virus 1 (HSV-1), cytomegalovirus (CMV), and EBV, while these motifs were rarely homologous to the components of measles virus, rubella virus, and varicella zoster virus. As for putative myelin autoantigens in MS, the motif of myelin basic protein was detected in only one patient (MS5). In patient MS6, identical peptide motifs homologous to the components of EBV were repeatedly detected from the 3 CSF samples (Table 3). The Expect value (*E*) is a parameter that describes the number of hits one can “expect” to see by chance when searching a database of a particular size. The lower the *E*-value, or the closer it is to zero, the more “significant” the match is. The *E*-values of the three peptide motifs SPxxMH, DPYQxP, and PYxxYQxP for EBV were 0.32, 0.008, 0.028, respectively, when we used BLAST limiting the organism to viruses.

## 4. Discussion

Using molecular immunologic strategies, there have been several analyses of intrathecally synthesized oligoclonally expanded immunogloblins with high affinity to identify specific antigens. The screening of libraries expressing protein products derived from chronic MS plaque messenger RNA with antibodies purified from plaques, CSF, or serum of patients with MS has not thus far revealed the antigenic targets of the MS antibody response [2].

The phage display method utilizes random peptide libraries displayed on phages expressing a large collection of peptide sequences (10^8^ or more) that mimic linear or conformational epitopes of folded protein domains, and even carbohydrate structures [5–7]. We analyzed the binding motifs of IgG in the CSF of HAM/TSP patients using the phage library method [3]. As a result, sequences highly homologous to HTLV-I gp46 were the common linear epitopes of high affinity-IgG exclusively detected in both CSF and sera of the patients. 

A few laboratories have previously reported the selection from RPL of several peptides that bind to antibodies present in the CSF of Caucasian MS patients. However, the results are still inconsistent. Rand and colleagues reported that CSF IgG antibody selected the amino acid sequence motif RRPFF from a random peptide library in five out of 14 American patients with MS. The RRPFF motif is found in EBV nuclear antigen [8]. Cortese and colleagues carried out a similar study of CSF from Italian patients with MS using the phage display method. Three different candidate amino acid motifs selected by CSF from two patients with MS were tested and, whereas they were recognized with equal frequency by serum from both MS and control patients, CSF from only 4 out of 55 patients with MS reacted with one of these three motifs. With another experiment, they concluded that CSF-enriched antibodies did not share specificities among MS patients [9, 10]. We tried to find a common motif with a 12-mer peptide library in a larger cohort than those in the previous studies.

After confirming that the phage display method combined with MEME and BLAST search could detect the target antigen of CSF IgG from HAM/TSP patients, we investigated the potential target antigens recognized by high affinity IgG in MS CSF using the phage display method. However, we could not detect a peptide sequence or antigen that is recognized exclusively by CSF IgG from most of MS patients. 

One possible reason that we could not detect an epitope is that the MS CSF IgG may recognize a conformational epitope or nonprotein antigens. Another possible reason is that MS CSF IgG may recognize epitopes with lower affinity that may not exist dominantly compared to HAM CSF IgG. In our study, CSF IgG from two MS patients with OCB could not recognize common peptide motifs. When exploring MS CSF IgG epitopes using the phage display method, it might be necessary to sequence much larger numbers of phage clones and to increase the number of pannings. It is still difficult to conclude that the MS CSF IgG antigen does not exist. However, considering the fact that no MS antigen has been determined so far, MS CSF IgG may not recognize a single MS antigen, differing from HAM/TSP CSF IgG which recognizes HTLV-I. Also, the fact that the common peptide motifs detected in MS and control groups were not significantly different may suggest that MS CSF IgG may recognize ubiquitous antigens, also differing from the case of HAM/TSP CSF IgG. 

Interestingly, in patient MS6, identical peptide motifs homologous to the components of EBV were repeatedly detected from the 3 CSF samples. The *E*-values of the three peptide motifs from MS-6 for EBV were 0.008–0.32. In the former study, the *E*-values of the common peptide motifs from HAM/TSP CSF IgG for HTLV-I were 0.15–0.95. The peptide motifs were indicative of the EBV specificity. MS CSF IgG may recognize antigens persistently and epitope spreading may not occur in some patients. 

Recently, Meinl and colleagues reported that inflammation in the central nervous system (CNS) provides a unique, B-cell-friendly environment, in which B lineage cells, notably long-lived plasma cells, can survive for many years. These long-lived plasma cells may produce oligoclonal IgG bands. CNS resident cells (mainly astrocytes and microglia) produce mediators such as chemokines, cytokines, and adhesion molecules that promote the local survival of plasma cells [11]. Whether the antibodies derived from these long-lived plasma cells target specific antigens or nonspecific ubiquitous antigens remains to be determined. 

MS CSF IgG is considered to be oligoclonally increased, and the factor that evokes this response may not be a single, rare MS antigen. Other factors that survive specific B cells may evoke this response. Such factors, rather than a single rare antigen, may be common in the pathogenesis of MS. Further analysis of such factors may be critical to understanding the pathogenesis of MS.

## Figures and Tables

**Table 1:** Common peptide motifs in MS patients.

MS1	MS2	MS3	MS4	MS5	MS6.1	MS6.2	MS6.3	MS7	MS8	MS9	MS10	MS11	MS12	MS13	MS14	MS15
[PHSRPPTK]	[IRLPH]	[MIKA]	[QPEY]	[HHPAHG]	[DPYQ]	[DPYQLAWE]	[DPYQxP]	[PAATARQH]	[NPIEKYL]	[KRTRINK]			[FFSxSP]	[WHDA]	[WNEPDWLK]	[HNAL]
PHMRPDTK	IRLPH	MNKT	QPEY	HHPAHG	DPYQ	DYYQQHWE	DPYQVP	PATTAYEH	NPASKYL	HRTRNNK			FYSMDP	WHDM	WYQPDWIQ	HNAL
PHLRPATK	IQLPH	MIKH	QPQY	HHPAHG	DPYQ	DPYQLPFD	EFYQYP	PAPTASDH	NPVLWYL	KRTRKSK			FFSNNP	WHDA	WPEPDWLK	ENAL
EHMRPALK	ISLPH	MSKA	QPMY	HHPAHQ	DPYQ	AYYQVPWE	DPYQLP	PAPTAEQS	NPASAYL	KRIMINK			FFRRDP	WHEL	WSNPDWLF	HIAL
YHSRPPMK	IALPH			HHPAHN	DQYQ	DPYQTAFF	GPYQDP	PAITASES	NPIDTHL				FYTMDP	WHSV	WNHKDILK	
PHMKPFQK	IRFPH			HHPAHM		DPWXIAWT	AFYQQP	PAATARQT	NPSETYV				FFRESP			
KHARPMLK	VRLPH					GAYQLAWA	IPMQKP	PALTAVQM	NPIETLL				FFTNNP			
PHRPPMTK	VSFPH						LNYQHP	PYATNRFH	NPSGYYL				FFSPSP			
PHSRPLPK	ITLPX								NPINSYL				FFSPSP			
LHXRPPTK	YRLPH						[SPPXMH]		NPLEHFF				FFSSSP			
PHRTPQAK	VTLPD								NPVDMHM				FFRPSP			
KHLPAPTK	QQLPH						SPPKMH		NPILHWL				FFRSSP			
YHSRGFPL							SPRNMH		NPITQLL				FFTDSP			
	[NPVEF]						SPVHMH		NPIYRYI				FFRSSP			
							SPPLMH		NPAYYLL				FYWPDP			
	NPIEW						SPKSMH		NPLLKHL				FFSHEP			
	NPVEF						FSPRMH		NPLEPML				FYSLSP			
	NPVEN								NLGTMWL				FFHVSP			
									GPTHKDV				FLSNTP			
									GPIHKDR				FLSSNP			
													FMSRTP			
													YFSTTP			

Parentheses indicate common peptide motifs in an MS patient.

Peptide sequences derived from random peptide library are listed below.

MS6.1, 6.2, 6.3 indicate 3 different samples from MS patient 6.

**Table 2:** Common peptide motifs in control group.

Cont 1	Cont 2	Cont 3	Cont 4	Cont 5	Cont 6	Cont 7	Cont 8	Cont 9	Cont 10
	[IHHPAHG]	[NPIEAF]	[KPPNP]	[GVTDY]		[HILHNPF]	[GPALSxSE]	[KPPNPS]	[IYPTTLPW]
	IHHPAHG	NPVENF	KPPNP	GVQDY		HILHNPF	RPALSMYE	KPPNPM	IYRTTLPW
	MHHPAHQ	NHCCAF	KPPNP	GVHDY		HSLHNPF	RPALSMYE	KPPNPM	IYPTTLPW
	SHHPAHG	NPIELW	KPPNP	GMTDY		HLLHSPF	MPALSVSE	KPPNPS	IYTVTLPW
	NHHPAHM	NPIVNY	KPPNP	GLIDY		HYAHNPF	GPALSHSE	KPPNPS	
		NPVLAW	KPPNP	QITDY		HNHHNPF	RPALTPQE	KPPNPS	[YLDL]
	[YLPK]	NPIEKL	KPPNP			HIMHDPL	QPALSSTE	KPPNPE	
		NPVLTF		[LPHWSP]		HNLHSPW	MPMLSAEE	KPPNPT	YYDL
	YLPK		[SLQSLRAAAM]				SPHISNKE	KPPNPL	YLDL
	YEPQ			LPHWSP			GPSLLNVE		YPDL
	YLSK		SIQSLRTAFM	LPHWSP			TPALHRLE		
	YLPT		SLQSLRGASA	LPHWMP			VPTLNVAE		
			SLSSLRAAAF	LPHWQP			GPSLHPSE		
			SVRSLHERVM	LPHWSF			MPGLPSQE		
				IRHWVP			SPATYFLE		
							SPQLSAHG		
							[PWSK]		
							PWSK		
							PWSK		
							PWSK		
							PWSK		
							PWSK		
							PWSK		
							PWSK		
							PWSK		

Parentheses indicate common peptide motifs in a control case.

Peptide sequences derived from random peptide library are listed below.

**Table 3:** Common peptide motifs derived from 3 CSF samples of MS6.

	Peptide motifs		
	(SPxxMH)	(DPYQxP)	(PYxxYQxP)
1999.12 (Remission)	**SP**PK**MH **	**DPYQ**V**P **	**PY**EF**YQ**Y**P **
	**SP**KS**MH **	G**PYQ**D**P **	**PY**LN**YQ**H**P**
	**SP**PL**MH **	**DPYQ**L**P**	
	**SP**RN**MH **		
	**SP**VH**MH**		

2000.1 (Relapse)	**SP**RID**H **	**DPYQ**L**P**	**PY**TG**YQ **
	**SP**PL**MH**	** DPYQ**SL	**PY**AG**YQ**
		**DPYQ**VY	
		**D**Q**YQ**Q**P**	

2000.10 (Remission)	**SP**YHS**H **	**D**Y**YQ**QH	**PY**SP**YQ**L**P **
	**SP**RN**MH**	**DPYQ**TA	**PY**NL**YQ**T**P**
	**SP**GN**MH**	**DPYQ**L**P**	T**Y**GA**YQ**LA
			**PY**AH**YQ**PY

EBV component	IYY**SP**SI**MH**R	P**DPYQ**L**P**FA	A**PY**QG**YQ**E**P**P
	(EBV VP19)	(EBV ZEBRA)	(EBV EBNA 6)

Bold-faced letter indicates common peptide motifs.
